# Developmental coordination disorder subtypes also vary in the pattern of behavioral and emotional problems

**DOI:** 10.3389/fpsyg.2024.1418295

**Published:** 2024-11-28

**Authors:** M. M. Schoemaker, J. M. Lust, B. Steenbergen, S. Houwen, J. E. M. Diepstraten, P. H. Wilson, M. Poelma

**Affiliations:** ^**1**^Department of Human Movement Sciences, University of Groningen, University Medical Center Groningen, Groningen, Netherlands; ^2^Behavioural Science Institute, Faculty of Social Sciences, Radboud University, Nijmegen, Netherlands; ^3^Faculty of Behavioural and Social Sciences, Inclusive and Special Needs Education Unit, University of Groningen, Grote Rozenstraat, Groningen, Netherlands; ^4^Rehabilitation Medicine, Sint Maartenskliniek, Hengstdal, Netherlands; ^5^Development and Disability Program, Healthy Brain and Mind Research Centre, Australian Catholic University, Melbourne Campus, Melbourne, VIC, Australia

**Keywords:** developmental coordination disorder, internalizing problems, externalizing problems, social problems, attention problems, subtypes

## Abstract

**Background:**

Behavioral and emotional problems in children with Developmental Coordination Disorder (DCD) are well documented. However, the heterogeneity of this group has been largely overlooked. Addressing this gap is important to develop individually-tailored interventions.

**Aims:**

Our three aims were to assess: (i) behavioral and emotional problems in children with DCD; (ii) behavioral and emotional problems in subtypes of DCD, and (iii) the context-specificity of these problems (home/school).

**Methods and procedure:**

Data were extracted from the medical records of a large sample of 93 children with DCD (79 boys, mean age 8.3) referred to a rehabilitation center. Behavioral and emotional problems were assessed with the Child Behavior Checklist (CBCL) and the Teacher Report Form (TRF).

**Outcomes and results:**

Two-third of the children presented with behavioral and emotional problems according to both parents and teachers. The subtypes with generalized motor problems were most affected, while the subtype with gross-motor problems was least affected. Children presented with more problems at home than at school.

**Conclusion and implications:**

Given the frequent occurrence of behavioral and emotional problems, clinicians should tailor their interventions to these problems in DCD. Knowledge of subtypes can inform these decisions.

## Highlights

- Two-thirds of children with DCD faced behavioral and emotional problems.
- Attention problems were the most often reported behavioral problem by parents.
- Internalizing problems were the most often reported emotional problem by teachers.
- Children with generalized motor problems faced the most co-occurring problems.
- Parents reported more behavioral and emotional problems than teachers.

## 1 Introduction

About 5 percent of primary school children have problems with the coordination of daily life motor tasks, while there is no medical condition explaining these coordination problems (Blank et al., 2019). These children are diagnosed with Developmental Coordination Disorder (DCD) when they meet the diagnostic criteria for this disorder as listed in the Diagnostic and Statistical Manual of Mental Disorders, operationalized in the clinical practice guideline for DCD (American Psychiatric Association, 2013; Blank et al., 2019). There are strong indications that children with DCD are also at risk of co-occurring behavioral and emotional problems that impact their well-being (Blank et al., 2019). Only a few studies, however, have examined the nature and frequency of the whole spectrum of behavioral and emotional problems within one sample (e.g., King-Dowling et al., 2015; Tseng et al., 2007). In addition, virtually none have addressed the question of whether the profile of these problems differs in different subtypes of DCD. To fill this knowledge gap, the present study examined the nature, frequency, and co-occurrence of behavioral and emotional problems in a clinical sample of children with DCD (reported in Lust et al., 2022) in both the home and the school context and explored differences regarding the nature and frequency of the emotional and behavioral problems within DCD subtypes as identified in Lust et al. (2022). As the motor skill problems of children with DCD are initially the most visible symptom, a significant number of behavioral and emotional problems co-occur (Green et al., 2006), such as social problems (Piek et al., 2005; Poulsen et al., 2008; Schoemaker et al., 2013), internalizing problems, such as anxiety and depression (Omer et al., 2019), externalizing problems (or behaviors that are harmful and disruptive to others), and attention problems (Dewey et al., 2002).

The link between DCD and internalizing symptoms is explained in the environmental stress hypothesis (ESH; Cairney et al., 2013). According to the ESH, the motor problems of children with DCD act as a primary stressor through which a child is exposed to secondary stressors, such as peer problems, lack of perceived social support, and reduced self-esteem. These stressors act as mediators in the development of internalizing symptoms (Gasser-Haas et al., 2020; Mancini et al., 2019; Wagner et al., 2016). Internalizing symptoms cover two dimensions: loneliness/social withdrawal and anxiety/ depression (Gresham et al., 1999). A higher number of internalizing symptoms has been reported in children with DCD compared with age-matched typically developing (TD) peers, as summarized in several reviews (Caçola, 2016; Draghi et al., 2019; Mancini et al., 2016, 2019; Missiuna and Campbell, 2014; Omer et al., 2019;). The extent to which children with DCD demonstrate more symptoms in one of both dimensions, however, varies across studies. King-Dowling et al. (2015) only found symptoms of social withdrawal in 4-year-old children with DCD, but no signs of anxiety/depression. In contrast, Piek et al. (2010) found more symptoms of anxiety/depression in children aged 6–12 years at-risk for DCD compared to TD children, but not social withdrawal.

Of the few studies that have investigated externalizing symptoms (aggressive and rule breaking behavior) in DCD, higher levels have been reported in these children relative to TD children (Dewey et al., 2002; Kanioglou et al., 2005; Tseng et al., 2007; Wagner et al., 2012). Intriguingly, symptoms of aggressive and rule-breaking behavior in DCD appear to vary between cultures. In two Canadian studies, aggressive behavior was notable in DCD relative to TD (Dewey et al., 2002; King-Dowling et al., 2015), whereas rule breaking behavior occurred more often in Taiwan (Tseng et al., 2007). As well, externalizing problems and hyperactivity and inattention, may mediate the relationship between motor proficiency and internalizing problems (de Medeiros et al., 2022). For example, one study has shown a stronger relationship between peer problems and externalizing symptoms than internalizing (Wagner et al., 2012).

Finally, one of the most often reported co-occurring problems in DCD has been poor attention, with a prevalence rate of 50% or higher for attention deficit hyperactivity disorder (ADHD; Blank et al., 2019; Green et al., 2006; Kaiser et al., 2015). Attention difficulties can compromise peer relationships (Wehmeier et al., 2010). And, because peer problems can mediate the relationship between motor proficiency and both internalizing problems (Mancini et al., 2019) and externalizing problems (Wagner et al., 2012), attention problems are likely to co-occur with social, internalizing and externalizing problems in children with DCD.

The heterogeneity of DCD as a disorder of movement has been recognized internationally (Blank et al., 2019). Several studies have considered subtypes in DCD, but the results are hard to compare due to differences in tests included in the cluster analysis and differences in samples (Macnab et al., 2001; Vaivre-Douret et al., 2011). Despite this, in a recent study, we identified four subtypes within a group of (clinically diagnosed) children with DCD (Lust et al., 2022) based on motor, visuo-motor and cognitive functioning that were largely in line with earlier studies. These sub-types differed in the severity and broadness of their motor problems. Specifically, we identified two subtypes with generalized motor problems but of differing severity, one subtype with gross motor problems primarily, and one subtype with fine-and visuo-motor problems. So far, the relation between specific subtypes of DCD and behavioral and emotional problems has not been addressed in previous studies. In light of the ESH and possible range of co-occurring behavioral and emotional problems, we need to better understand whether these co-occurring problems vary with DCD subtype. The ESH states that poor motor performance is the primary stressor, which eventually leads to internalizing problems. Whether we can extend this argument to every subtype of DCD remains uncertain.

Another important consideration is whether the presence of specific behavioral and emotional problems in DCD is context-specific. So far, to our knowledge only two studies compared problem areas in DCD across settings with both parents and teachers reporting behavioral problems. Only parents reported fewer adaptive skills for the DCD group in the first study (Davis et al., 2007), whereas more signs of hyperactivity and more prosocial behavior were reported by parents compared to teachers in the second study (Crane et al., 2017). Indeed, correlations between parent (home and community setting) and teacher (school setting) ratings are generally significant but low, with parents reporting more behavior and emotional problems than teachers (Huang, 2017). This stresses the need to include cross-informant information from different settings when studying co-occurring behavioral and emotional problems in DCD.

### 1.1 Present study

To the best of our knowledge, no study has yet investigated the full spectrum of possible co-occurring behavioral and emotional problems in a large clinical sample of children diagnosed with DCD (Lust et al., 2022). Also, existing studies tend to focus on the occurrence of these problems in one specific context, such as the home or school (King-Dowling et al., 2015; Van den Heuvel et al., 2016). This may distort our understanding of the generality versus specificity of these problems. In the present study, we took both contexts, home and school, into account, using different informants, i.e., parent and schoolteacher. The *aim* of our study was therefore threefold. First, to assess the nature, frequency and co-occurrence of behavioral and emotional problems in 118 children clinically diagnosed with DCD according to DSM-5 Criteria (American Psychiatric Association, 2013). More behavioral and emotional problems were expected in our sample of children with DCD compared to a normative sample of TD children, in line with previous research. Second, to examine differences in the nature, frequency and co-occurrence of behavioral and emotional problems in the four DCD subtypes that were found in a recent study (Lust et al., 2022). Since these subtypes differed in their motor proficiency across different skill domains and cognitive ability, we expected that the two subtypes with more generalized and severe motor problems would demonstrate more behavioral and emotional problems than the two subtypes with more specific motor problems (e.g., either fine-or gross-motor). Third, to explore differences in the nature and frequency of behavioral and emotional problems across settings, i.e., home (parents) and school (teachers). We expected parents to report more behavioral and emotional problems than teachers, in line with previous research (Huang, 2017). Data from two questionnaires from the Achenbach System of Empirically Based Assessment (ASEBA) were used from the sample described in Lust et al. (2022), the Child Behavior Checklist (CBCL, completed by the primary caregiver) and the Teacher’s Report Form (TRF; Achenbach and Rescorla, 2001).

## 2 Methods

### 2.1 Participants

This study is a retrospective medical record study of children referred to ZOOM-IN, an expert center of a rehabilitation clinic in Ubbergen, Netherlands. See Lust et al. (2022) for detailed information about this sample. Children referred to ZOOM-IN have problems with everyday motor tasks which affects participation at home and at school. Referred children enter a two-day screening to assess their motor functioning, communication, neurodevelopmental functioning, cognitive level, and behavior. Assessments are performed by a rehabilitation physician, a physical therapist, an occupational therapist, a psychologist and, on indication, a speech therapist. In addition, parents and teachers fill out questionnaires about the behavior of their child. After these 2 days, children may receive the diagnosis DCD if they meet the diagnostic criteria according to the DSM-5 (American Psychiatric Association, 2013) or earlier DSM-IV. Parents and teachers of all referred children are provided with advice to help the child in daily life. Between 2009 and 2018, 891 children were screened. Parents of these children filled out an informed consent to use the recorded data of their child for this retrospective study. In total, 379 parents gave permission. Only data of 5–12 year old children and with a confirmed diagnosis of DCD according to DSM-IV or DSM-V criteria were enrolled in the present study, yielding a total of 118 children. Of the 118, CBCL and TRF data of 93 children were available and included in the current study. Of these 93, data of 72 children were available for subtype analysis, as not all 93 children were included in the original cluster analysis due to missing data on indices for the cluster analysis (see Lust et al., 2022). Four subtypes were detected by Lust et al. (2022): Subtypes 1 ‘less severe generalized problems (*n* = 23)’ and 3 ‘severe generalized motor problems (*n* = 16)’ were characterized by below average performance on all motor subtests of the Movement Assessment Battery for Children-2nd Edition (MABC-2; Smits-Engelsman, 2010) and the visual perception subtest of the Beery-Buktenica Developmental Test of Visual-Motor Integration (Beery-VMI; Beery et al., 2010) as well as borderline performance IQ scores, with subtype 3 being the most affected with subtest scores on the MABC-2 below the 5th percentile; subtype 2 “gross motor problems (*n* = 19)’ mainly had problems with the gross motor subtests of the MABC-2, and subtype 4 ‘fine motor problems (*n* = 14)’ had lower scores on all tasks requiring fine motor skills of the MABC-2 and Beery-VMI and borderline performance IQ. Demographic (age, sex, education) and clinical characteristics (BMI, comorbidity, gestational age, birthweight) were not significantly different between the clusters. Ethical approval for the use of this clinical database for retrospective scientific research was granted by the local Ethics Committee of the Faculty of Social Science at Radboud University (reference: ECSW-2020-133) and the local ethics committee of the rehabilitation center (reference: 2018/06/20a/MVo/eb).

### 2.2 Procedure

Data from the medical records of these children were entered anonymously in a data file. Apart from behavioral data (CBCL and TRF), demographic information (age, sex, IQ, gestational age, birth weight, maternal education level, BMI), and the Movement ABC-2 score were also entered.

### 2.3 Materials

The Child behavior Checklist (CBCL 1.5–5; CBCL/6–18; Achenbach and Rescorla, 2001) was used to measure behavioral and emotional problems. Both the CBCL 1.5–5 and the CBCL 6–18 are filled out by the primary caregiver/parent. The CBCL 6–18 includes 118 items comprising eight syndrome scales (anxious/depressed; withdrawn/depressed; somatic complaints; social problems; thought problems (obsessive thoughts, tics, self-harm, compulsions, and hallucinations); attention problems (inattention, hyperactivity, impulsivity); rule-breaking behavior; aggressive behavior). The CBCL 1.5–5 includes 99 items, of which only the syndrome scales anxious/depressed, withdrawn/depressed, somatic complaints, attention problems and aggressive behavior were included in the present study. The first three syndrome scales make up the internalizing scale, while aggressive and rule-breaking behavior make up the externalizing scale. Each item is scored on a three-point scale (0 = not true to 2 = very true/often true). A *total problems score* is the sum of the 118 item scores. *Syndrome scale scores* are the sum of the individual item scores belonging to the respective scales. Raw scores were transformed into T-Scores and percentile scores. T-scores for syndrome scales below 67 are considered normal, T-scores 67 to 70 are subclinical (93–97^th^ percentile), and T scores above 70 are considered to be in the clinical range (> 98th percentile). For *composite scale scores*, T scores 60–63 are subclinical, and scores above 63 are in the clinical range.

The Teacher’s Report Form (C-TRF 1.5–5; TRF/6–18, Achenbach and Rescorla, 2001) was used to measure the behavioral and emotional problems of a child from a teacher’s point of view. The TRF 6–18 includes 118 items and covers the same syndrome scales as the CBCL. The C-TRF includes 99 items, and the same 5 syndrome scales as the CBCL 1.5–5 were included in the present study. Both the CBCL and the TRF are reliable and valid questionnaires (Achenbach and Rescorla, 2001). This also holds for the Dutch versions (Verhulst et al., 1996).

### 2.4 Data analysis

The data were checked for normality and homogeneity of variance. To test whether behavioral and emotional problems are more common in children with DCD than in the typical population from the perspective of the parents and teachers, we calculated the observed percentages of children with DCD in the normal, subclinical and clinical range for each CBCL and TRF category. Chi-square tests were performed to compare the percentage of children in the normal, subclinical and clinical range with the percentage of children expected in that range according to the norms for the syndrome and composite scales. To test the level of agreement between parent and teacher ratings for syndrome and composite scales, intra-class correlations (ICC) were calculated. Inter-rater reliability is poor when ICC values are smaller than 0.5, moderate when between 0.5 and 0.75, good between 0.75 and 0.9, and excellent when greater than 0.9 (Koo and Li, 2016). To examine differences between subtypes, the percentages of children scoring in the (sub)clinical range per syndrome scale per subtype were first calculated. Next, to explore whether the subtypes identified by Lust et al. (2022) differed with regard to the nature and frequency of behavioral and emotional problems, we compared the percentage of children in the (sub)clinical domain of the CBCL and TRF for each subtype with the expected percentage according to the norms, tested using Chi-square and a Bonferroni-adjusted alpha level of ≤0.002 (4 subtypes are compared to the norms on 6 outcome measures = 0.05/24 = 0.002). Lastly, the percentages of children per subtype with 0, 1, 2 or > 2 scores in a (sub)clinical range of the syndrome scales on the CBCL and TRF were calculated. Children with >2 scores in a (sub)clinical range were taken together due to the small number of children with 3, 4, 5, and 6 scores in a (sub)clinical category separately.

## 3 Results

### 3.1 Demographic characteristics of the DCD group

See Table 1 for the demographic characteristics. The boy: girl ratio was 5.6: 1.

**Table 1:** Demographic characteristics of the DCD sample.

		Range
Age, mean (sd)	8.3 (2.3)	5.1–15.6
Sex, *n* (%)		
Boys	79 (84.9%)	
Girls	14 (15.1%)	
Gestation *n* (%)^a^		
< 37 weeks	5 (6.8%)	
≥ 37 weeks	69 (93.2%)	
Birth weight *n* (%)^b^		
≤2,500 g	13 (15.3%)	
>2,500 g	72 (84.7%)	
Maternal education level *n* (%)^c^		
Low	4 (5.4%)	
Middle	39 (52.7%)	
High	31 (41.9%)	
Body Mass Index (BMI) *n* (%)^d^		
< 21 (healthy weight)	69 (89.6%)	
≥ 21 (overweight)	8 (10.4%)	
Co-occurring diagnoses *n* (%)		
ADHD	9 (9.7%)	
ASD	3 (3.2%)		Motor performance
MABC2^e^-Total (percentile)	4.1 (4.6)^f^	0.1–16.0
MABC2-Manual Dexterity^e^ (percentile)	10.4 (14.1)^f^	0.1–75.0
MABC2-Aiming and Catching^e^ (percentile)	19.1 (20.9)^f^	0.1–84.0
MABC2-Balance^e^ (percentile)	9.7 (13.2)^f^	0.1–75.0
IQ		
PIQ^g^, mean (sd)	89.8 (12.9)	66–126
VIQ^h^, mean (sd)	104.9 (13.2)	80–140
TIQ^i^, mean (sd)	97.2 (12.1)	74–133

a missing data 20.4%.

b missing data 8.6%.

c missing data 20.4%.

d missing data 17.2%.

e MABC2, Movement Assesment Battery for Children-2^nd^ Edition.

f Mean (SD) percentile score.

g PIQ, WISCR-III-NL Performal Intelligence Quotient.

h VIQ, WISC-III-NL Verbal Intelligence Quotient.

i TIQ, WISC-III-NL Total Intelligence Quotient.

### 3.2 Parent perspective of behavioral and emotional problems

Observed percentages for internalizing and externalizing problems, and for four out of eight syndrome scales: withdrawn-depressed, social, thought and attention problems were significantly higher than the expected percentages (see Table 2). Specifically, 33.3% of the children with DCD had internalizing problems (subclinical and clinical combined), 19.4% had externalizing problems, and 10.8% had both. The most frequently reported problem is attention problems (43.1%), followed by social problems (30%), withdrawn-depressed (23.7%), and thought problems (17.6%).

**Table 2:** Comparison of the expected and the observed division of children with DCD in CBCL and TRF categories normal, subclinical and clinical.

CBCL (*N* = 93)						TRF (*N* = 92)				
	Normal	Subclinical	Clinical	*Χ*^2^ (*df* = 2)	*p*	Normal	Subclinical	Clinical	*Χ*^2^ (*df* = 2)	*p*
CBCL/TRF % expected*	93%	5%	2%			93%	5%	2%		
Internalizing problems	66.7% (*n* = 62)	11.8% (*n* = 11)	21.5% (*n* = 20)	175.898	<0.001	69.8% (*n* = 64)	9.8% (*n* = 9)	20.4% (*n* = 19)	154.984	<0.001
Externalizing problems	80.6% (*n* = 75)	5.4% (*n* = 5)	14% (*n* = 13)	61.907	<0.001	76.1% (*n* = 70)	13% (*n* = 12)	10.9% (*n* = 10)	45.253	<0.001
Anxious	90.3% (*n* = 84)	5.4% (*n* = 5)	4.3% (*n* = 4)	2.047	0.359	82.6% (*n* = 76)	13% (*n* = 12)	4.3% (*n* = 4)	13.093	<0.001
Withdrawn	76.3% (*n* = 71)	12.9% (*n* = 12)	10.8% (*n* = 10)	44.416	<0.001	83.7% (*n* = 77)	10.9% (*n* = 10)	5.4% (*n* = 5)	10.544	<0.005
Somatic complaints	90.3% (*n* = 84)	6.5% (*n* = 6)	3.2% (*n* = 3)	0.747	0.688	95.7% (*n* = 88)	4.3% (*n* = 4)	-	1.3363^c^	0.248
Social problems^a^^,^^b^	70% (*n* = 56)	15% (*n* = 12)	15% (*n* = 12)	70.378	<0.001	71.1% (*n* = 54)	18.4% (*n* = 14)	10.5% (*n* = 8)	78.070	<0.001
Thought problems^a^^,^^b^	82.5% (*n* = 66)	6.3% (*n* = 5)	11.3% (*n* = 9)	25.615	<0.001	82.3% (*n* = 65)	7.6% (*n* = 6)	10.1% (*n* = 8)	19.877	<0.001
Attention problems	57% (*n* = 53)	28% (*n* = 26)	15.1% (*n* = 14)	172.863	<0.001	84.8% (*n* = 78)	9.8% (*n* = 9)	5.4% (*n* = 5)	8.525	0.014
Rule breaking^a^^,^^b^ behavior	95% (*n* = 76)	2.5% (*n* = 2)	2.5% (*n* = 2)	1.054	0.59	94.9% (*n* = 75)	5.1% (*n* = 4)	5.1% (*n* = 4)	.721^3^	0.396
Aggressive behavior	87.1% (*n* = 81)	7.5% (*n* = 7)	5.4% (*n* = 5)	5.591	0.061	89.1% (*n* = 82)	6.5% (*n* = 6)	6.5% (*n* = 6)	2.401	0.301

a *N* = 80 (missing data CBCL).

b *N* = 79 (missing data TRF).

c df = 1.

^*^Expected percentages according to norms CBCL/TRF for total, composite and syndrome scales.

### 3.3 Behavioral and emotional problems according to parents across subtypes

**Figure 1:**
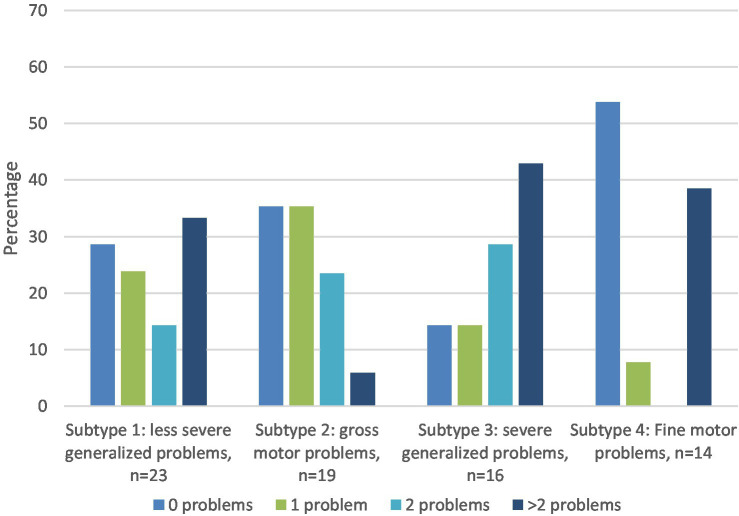
Percentage of children in each DCD subtype with 0, 1, 2, or more than 2 behavioral and emotional problems (scores in (sub)-clinical range of CBCL syndrome scales). All percentages above 25% are significantly higher than expected according to the norms.

Attention problems were reported significantly more often by parents across all subtypes than would be expected according to CBCL norms, with percentages ranging from 35.7 to 56.3%. Parents of children in subtype 1 (*n* = 23, less severe generalized motor problems) reported significantly more attention problems (47.8%), internalizing problems (43.5%), and social problems (38.1%). For subtype 2 (*n* = 19, primarily gross motor problems) significantly more attention problems were reported (42.1%). For subtype 3 (*n* = 16: severe generalized motor problems), significantly more attention problems (56.3%), internalizing problems (50%), and social problems (50%) were reported. For subtype 4 (*n* = 14, primarily fine motor problems), significantly more attention problems (35.7%), internalizing problems (28.6%), externalizing problems (28.6%), and social problems (30.8%) were reported. Overall, as a function of subtype (1, 2, 3 and 4), the percentages of children with one or more behavioral or emotional problem in the clinical range were 71.4, 64.7, 85.7, 46.2%, respectively (see also Figure 1; Appendix A).

### 3.4 Teacher perspective of behavioral and emotional problems

Comparable to parents, the percentages of children with DCD in the normal, subclinical and clinical range were significantly higher than normative values for internalizing problems and externalizing problems (Table 2). Significantly larger percentages of children with DCD were evident for 5 out of 8 syndrome scales: anxious-depressed, withdrawn-depressed, social problems, thought problems, and attention problems. The most frequently reported problem by teachers was internalizing problems (30.2%), followed by social problems (28.9%), externalizing problems (23.9%), thought problems (17.7%), and attention problems (15.2).

### 3.5 Behavioral and emotional problems identified by teachers across DCD subtypes

**Figure 2:**
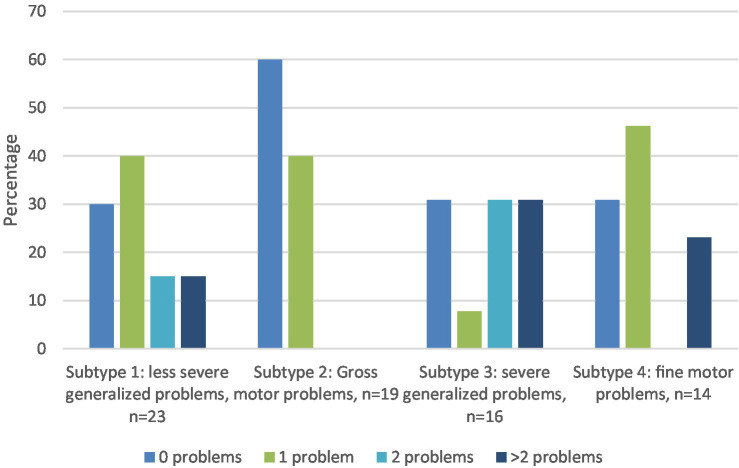
Percentage of children in each DCD subtype with 0, 1, 2, or with more than 2 behavioral and emotional problems (scores in (sub)-clinical range of TRF syndrome scales). All percentages above 25% are significantly higher than expected according to the norms.

Internalizing problems were reported significantly more often by teachers across all subtypes than would be expected according to CBCL norms, with percentages ranging from 28.6 to 35.7%. Teachers of children in subtype 1 (*n* = 23, less severe generalized motor problems) reported significantly more internalizing problems (30.4%), social problems (30%), and thought problems (28.6%). For subtype 2 (*n* = 19, primarily gross motor problems), significantly more internalizing problems were reported, only (33.3%). For subtype 3 (*n* = 16: severe generalized motor problems), more attention problems (31.3%), internalizing problems (37.5%), externalizing problems (37.5), and social problems (53.8%) were reported. For subtype 4 (*n* = 14, primarily fine motor problems), more attention problems (21.4%), internalizing problems (28.6%), externalizing problems (42.9%), and social problems (38.5%) were reported. Figure 2 shows, as a function of subtype, the percentages of children reported by teachers as having zero, one, two or more than two scores in a (sub)-clinical domain of the TRF. By DCD subtype, the percentage of behavioral or emotional problems in the clinical range as rated by teachers were 70, 40, 69.2, 69.2%, respectively (see also Figure 2).

### 3.6 Comparison of parent and teacher perspective

**Figure 3:**
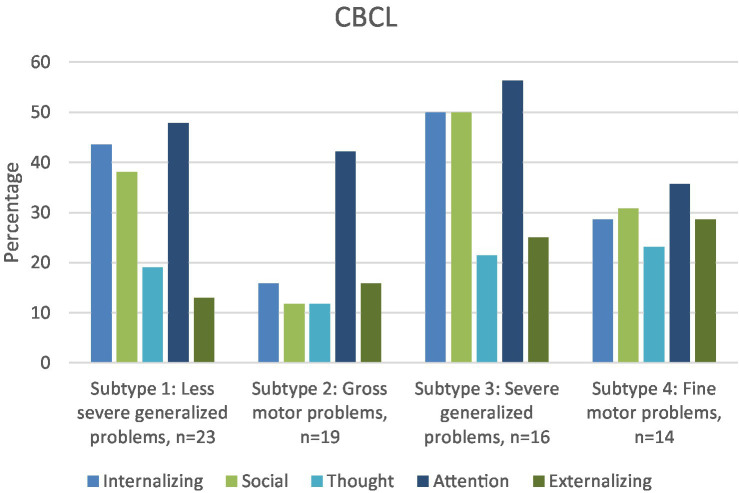
For each DCD subtype, percentage of children in the subclinical/clinical range of the internalizing, externalizing, social, thought and attention scales of the CBCL.

**Figure 4:**
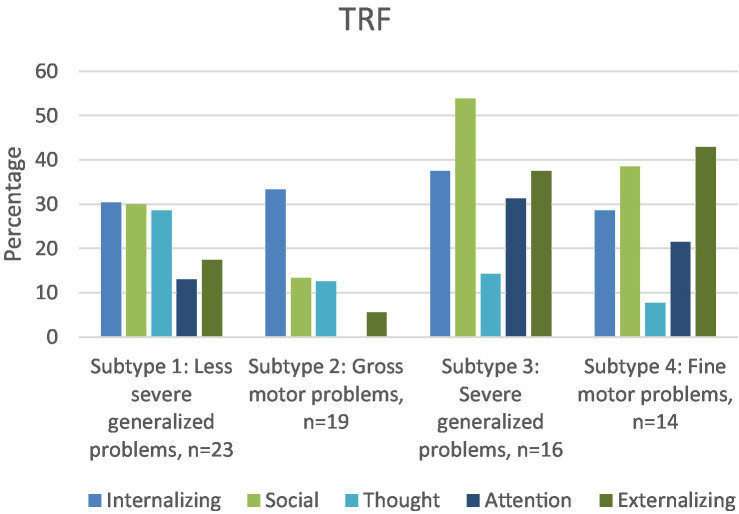
For each DCD subtype, percentage of children in the subclinical/clinical range of the internalizing, externalizing, social, thought and attention scales of the TRF.

As shown in Table 3, intraclass correlations between parent and teacher ratings are significant, but poor. According to Figures 3, 4, parents more often reported attention problems in the (sub)-clinical range. Both parents and teachers reported internalizing and social problems in the clinical range for subtypes 1, 3, and 4.

**Table 3:** Intra-Class Correlations between CBCL and TRF total problems scale, internalizing and externalizing scales and syndrome scales for the whole sample.

	ICC (range)	*p*
CBCL-TRF total	0.355 (0.153–0.521)	<0.001
Internalizing problems	0.341 (0.147–0.509)	<0.001
Externalizing problems	0.475 (0.279–0.604)	<0.001
Anxious	0.308 (0.115–0.480)	0.001
Withdrawn	0.362 (0.171–0.527)	<0.001
Somatic complaints	0.178 (−0.009–0.358)	0.023
Social problems	0.438 (0.24–0.601)	<0.001
Thought problems	0.250 (0.013–0.446)	0.013
Attention problems	0.299 (0.090–0.481)	<0.001
Rule breaking behavior	0.301 (0.085–0.480)	0.004
Aggressive behavior	0.461 (0.283–0.608)	<0.001

## 4 Discussion

The *first aim* of our study was to assess the nature and frequency of behavioral and emotional problems and their co-occurrence in a sample of 118 children clinically diagnosed with DCD. Our findings underline that children with DCD experience a higher rate of internalizing and externalizing problems, social, attention and thought problems. The *second* aim of this study was to assess whether the nature and frequency of behavioral and emotional problems is different for DCD subtypes (Lust et al., 2022). The results revealed that children with primarily gross motor problems were the least affected by these associated problems, both in nature and frequency, while those children with severe generalized motor problems were the most affected. The *third* aim of our study was to explore differences between parent (CBCL) and teacher (TRF) report of behavioral and emotional problems, with data suggesting a significant but weak relationship. This underlines the importance of including different informants in assessing the psychosocial well-being also of children with DCD.

### 4.1 Behavioral and emotional problems according to parent and teachers

Our results reveal both sobering and encouraging signs about the associated behavioral and emotional problems of children with DCD. On the positive side, DCD is a heterogeneous disorder also with regard to behavioral and emotional problems. Apart from subtype 3 about one third of our sample did not experience any behavioral or emotional problems according to their parents or teachers. In another recent study, only 24% of children with DCD did not show any behavioral or emotional problems (Pimenta et al., 2023), measured on the Strengths and Difficulties Questionnaire (SDQ; Goodman and Goodman, 2009). These findings highlight that some children with DCD have a degree of resilience that helps them cope with the consequences of their motor problems. At the same time, about two-thirds of the children with DCD do encounter behavioral and emotional problems. In about 20% of cases one co-occurring behavioral and emotional problem was reported. However, for about 40% of the children two or more co-occurring problems were reported.

#### 4.1.1 Social problems

According to both parents and teachers, more children with DCD (25%) scored in the (sub)-clinical range of the social domain than would be expected according to the norms of the CBCL and TRF (7%). These results corroborate previous results in elementary school-aged children with DCD (Chen et al., 2009; Crane et al., 2017; Tseng et al., 2007; van den Heuvel et al., 2016), but are not in line with the results of a group of a younger non-referred 4-year-old children suspected of having DCD (King-Dowling et al., 2015). It is possible that social problems are not yet present in 4-year-old children, but gradually develop as children increasingly participate in social contexts outside the family, such as sports and games. Supporting this is a narrative account of the social problems of 8–12 year old children with DCD in which exclusion and bullying were reported as a consequence of not being able to properly participate in sports and games (Zwicker et al., 2018). Also the current clinically referred group may have been facing more severe motor coordination difficulties than the non-referred sample of King-Dowling et al. (2015). More severe motor coordination difficulties have been found to be related to a higher incidence of co-occurring problems (e.g., Schoemaker et al., 2013).

#### 4.1.2 Internalizing problems

Internalizing problems were present in about 30% of our sample according to both parents and teachers. An increased risk of internalizing symptoms in children with DCD is a common finding in the literature irrespective of the nature of the sample (population-based or clinically-referred) or respondent (parent, teacher or self-report; See Omer et al., 2019 for a review). The percentage of 30% is in line with the results of Pimenta et al. (2023; 32.7%), but lower than the 50 and 55% reported in the studies by Emck et al. (2009) and Tseng et al. (2007), measured with the CBCL and both including children with DCD screened from elementary schools. However, the percentage is much higher than the 13% internalizing problems obtained in population-based studies of children in the Netherlands (Van den Heuvel et al., 2016; Van der Ende et al., 2016). Only a few studies addressed the question about which syndrome scales belonging to the internalizing subscales of the CBCL and TRF (i.e., anxious/depressed symptoms, withdraw/depressed symptoms and somatic complaints) differed between children with DCD and TD children. These studies reported mixed results in the primary school age range. Specifically, two studies investigating parent-reported behavioral and emotional symptoms found significantly more signs of both withdrawn/depressed and anxious/depressed behavior in children with DCD compared to TD children (Emck et al., 2009; Tseng et al., 2007), whereas one study only reported significantly more signs of withdrawn/depressed symptoms (Chen et al., 2009), even though the samples (DCD children selected from elementary schools) and measures used (CBCL) were the same. The only study investigating teacher reports found significantly more signs of both withdrawn/depressed and anxious/depressed symptoms (Van den Heuvel et al., 2016). In our study parents mainly reported symptoms of withdrawn/depressed behavior, whereas teachers reported symptoms of both anxious/depressed together with withdrawn/depressed symptoms. Finally, somatic complaints were not overrepresented in our DCD sample according to both parents and teachers, which is in line with previous studies (Chen et al., 2009; Emck et al., 2009; Tseng et al., 2007; Van den Heuvel et al., 2016).

#### 4.1.3 Externalizing problems

Externalizing problems were significantly more often reported than expected according to the CBCL and TRF norms (19.4% as reported by parents; 23.9% by teachers), and slightly higher than the 15% found in a population-based sample of children in the Netherlands (Van der Ende et al., 2016). Moreover, externalizing problems were less common than internalizing problems in our study, which is in line with earlier studies (Emck et al., 2009; Tseng et al., 2007). In our sample, aggressive behavior contributed most to the reported externalizing problems, while rule-breaking behavior was not overrepresented, in line with previous research (Dewey et al., 2002; King-Dowling et al., 2015). Aggressive behavior in DCD may be a consequence of the frustration that children feel when they struggle to perform motor skills, and the negative reaction of peers; however, this remains to be examined.

#### 4.1.4 Attention problems

Similar to other studies, attention problems were the most frequently reported co-occurring behavioral problem by parents in our sample. The attention problem scale covers signs of inattention, hyperactivity and impulsivity (Achenbach and Rescorla, 2001), and scores in the clinical range are a predictor of ADHD (Raiker et al., 2017). Generally, a prevalence rate of 50% is reported for ADHD in children with DCD in literature (Green et al., 2006; Kaiser et al., 2015). Parent reports in our study approached this figure (43.1%; see also Pimenta et al., 2023), while teacher reports were substantially lower at 15%. Among children with ADHD, parents commonly report more attention problems than teachers (Narad et al., 2015; Kennerley et al., 2016). The school environment offers more structure than the home environment, which elicits less hyperactive or impulsive behavior. It would be interesting to investigate if a more structured home environment prevents or attenuates the occurrence of attention problems in DCD. Interestingly, a new finding from our study is that attention problems often co-occurred with social problems. Children with ADHD are known to have social problems. About 50% have problems with peer relationships, which may be a consequence of their impulsive behavior and inability to cooperate with other children (Wehmeier et al., 2010). Based on the combined presence of DCD and attention problems it may be interesting to investigate if attention problems exacerbate the social problems.

#### 4.1.5 Thought problems

A problem area less addressed in the DCD literature is the occurrence of thought problems, which includes obsessive thoughts, tics, self-harm, compulsions, and hallucinations (Achenbach and Rescorla, 2001). In the present study, both parents and teachers reported a significantly higher percentage of thought problems relative to normative data (17.6 and 17.7% respectively, compared with 7% according to norms). Thought problems are a significant predictor of Autism Spectrum Disorder (ASD), particularly when they are combined with high scores on the withdrawn-depressed and social problems subscales (Biederman et al., 2010; Ooi et al., 2011). According to Hoffmann et al. (2016), high scores on thought problems, social problems, withdrawn/depressed behavior and attention problems can differentiate children with high functioning autism from those without. In the present study, 10 out of 13 children with thought problems also scored in the clinical range on the attention subscale, and 8 out of these also scored in the (sub)-clinical range on withdrawn/depressed behavior and/or social problems. Therefore, it is fair to conclude that about 15% of the children in our sample presented with autistic symptoms. The co-occurrence between DCD and ASD is well documented, with rates of co-occurrence between 25 and 90% reported in samples of children with ASD (Kopp et al., 2010; Bhat, 2020; Miller et al., 2021). Consequently, clinicians should take steps to assess for possible co-occurring ASD when assessing children with DCD.

### 4.2 Behavioral and emotional problems across subtypes

To our knowledge, this is the first study to investigate the types of behavioral and emotional problems present in different (clinically-derived) DCD subtypes, based specifically on motor and cognitive functioning and visuo-motor integration (Lust et al., 2022). Attention problems at home were a feature of all subtypes, as revealed by a significantly higher percentage of attention problems reported by parents (35.7–56.3%) compared with CBCL norms (7%). In the school context, only parents of children with the most severe generalized problems (subtype 3) reported more attention problems. Internalizing and social problems were significantly more often present in the subtypes with generalized motor problems (subtypes 1 and 3) and the subtype with primarily fine-motor problems (subtype 4) according to both parents and teachers compared with norms. The co-occurrence of internalizing and social problems is in line with one of the assumptions of the *environmental stress hypothesis* (ESH, Cairney et al., 2013) which states that social problems may mediate the presence of internalizing problems (Gasser-Haas et al., 2020; Mancini et al., 2019; Wagner et al., 2016).

Parents of the children within the two subtypes with generalized motor problems (subtypes 1 and 3) reported the highest percentage (respectively 71.4 and 85.7%) of children with one or more behavioral and emotional problem, in particular internalizing, social and attention problems. The more severe the motor problems (subtype 3), the higher the percentage of children with behavioral and emotional problems. In addition to internalizing, social and attention problems, children within subtype 3 also had a higher percentage of externalizing problems, particularly in the school context. As children with severe generalized motor problems (subtype 3) fail in both gross-and fine-motor tasks, they are likely exposed to all of the stressors included in the ESH, such as low perceived competence, and lack of social support, with both internalizing and externalizing problems as a result.

Remarkably, the subtype with primarily gross-motor problems stood out, as internalizing problems were their only problem according to teachers, and attention problems their only problem according to parents. As far as we know, only one previous study focused on behavioral and emotional problems in children with gross-motor problems (Emck et al., 2009), in which high percentages of emotional and behavioral problems were reported. However, Emck et al. (2009) only assessed gross-motor performance of the children, so fine-motor problems cannot be ruled out. This hampers comparison between the results of Emck et al. (2009) and our study. Still, the question remains as to why internalizing problems were only reported in the school context in our subtype with primarily gross-motor problems. A possible explanation might be that many activities of daily living at home involve fine-motor tasks. Failure at these tasks may regularly result in negative feedback from parents. By comparison, if only gross-motor problems are present, children may experience less negative feedback while negotiating day-to-day activities in the home. Consequently, these children may be less exposed to the stressors known to increase emotional problems according to the ESH, such as lack of support from parents. In the school context however, they cannot avoid gross-motor activities, especially during physical education and school breaks; hence, they may be more exposed to stressors such as reduced support from teachers and peers which can evoke internalizing problems (withdrawn/depressed behavior). These possible (causal) explanations warrant further study, in order to confirm the generalizability of our results to other populations of children with mainly gross-motor problems.

Apart from this explanation, one might also argue that less behavioral and emotional problems in the subtype with gross motor problems may be related to the higher performal IQ scores in this subtype. Children in subtypes 1, 3, and 4 have lower performal IQ scores than those in subtype 2. However, these lower performal IQ scores are likely to be due to the fine motor problems present in subtypes 1, 3, and 4, but not in subtype 2. Several performal IQ subtests require fine motor skills, and lower levels of fine motor skills affect performal IQ test results. As mean total IQ scores are in the normal range for all 4 subtypes, it is unlikely that lower IQ is related to the increased prevalence of behavioral and emotional problems in subtypes 1, 3, and 4.

Subtype 4 concerns children with primarily fine-motor problems, poor balance, and visuo-motor integration problems. According to parents, about 50% of the children within this subtype do not have any behavioral or emotional problems. However, the children within subtype 4 who do have problems are likely to have a cluster of more than 2 problems (38%), in particular internalizing and externalizing, social and attention problems. In the school context, the same picture is revealed, but the percentage of children with more than 2 problems is smaller (22%). It is unclear why children within this cluster either have no problems, or several. As the number of children in this subtype was rather small (*n* = 14), future studies should investigate whether this mixed pattern of co-occurring problems is a recurring one.

Our results highlight the heterogeneity of DCD in the behavioral and emotional domain, as children differ in both the nature and frequency of the problems they face within the different subtypes. In addition, their problems can be context-specific, as some problems are visible in the home context, but not in the school context, and vice versa. The clinical implications of our study are quite clear: assessment of children with DCD needs to be stretched beyond the motor domain considering the risk of behavioral and emotional problems, particularly in children with generalized motor problems. Early identification of co-occurring behavioral and emotional problems can lead to interventions that prevent these problems becoming lifelong conditions. According to the ESH, several factors mediate the occurrence of mental health issues (in particular internalizing problems), such as lack of self-esteem, lack of social support, and lack of communication skills (Omer et al., 2019). On the other hand, the presence of high self-esteem, good social communication skills, high IQ and the absence of bullying have been identified as protective factors that make children more resilient to the challenges posed by their motor problems (Lingam et al., 2012). The present study was not designed to shed light on possible protective factors. However, the finding that one third of our group of clinically-referred children did not encounter emotional and behavioral problems stresses the need to investigate in-depth those factors that build resilience.

### 4.3 Comparison of parent and teacher perspective

Correlations between parent and teacher reports on the different domains were significant, but low-moderate, ranging from 0.17 for somatic complaints to 0.48 for externalizing behavior. The low agreement between parents and teachers on the CBCL and TRF is well documented, as a meta-analysis of 269 data samples revealed an average correlation of 0.28 between parents and teachers, whereas the correlation between pairs of parents was much higher (0.60; Achenbach et al., 1987). These results again stress that the behavior of a child is context specific. Children demonstrate different behaviors in the home and school environment by virtue of the types of environmental and task structures imposed, resulting in low correlations between both contexts (De Los, 2013). These results stress the need to collect reports from informants in multiple contexts to get a comprehensive picture of a child’s emotional and behavioral problems.

### 4.4 Limitations and strengths

A first strength of our study is the inclusion of a relatively large group of clinically referred children with DCD including the comprehensive assessment these children underwent (as described in Lust et al., 2022), and having both parents and teachers as informants. Secondly, no other studies compared emotional and behavioral problems across subtypes of DCD, with subtyping based upon their motor and cognitive functioning. A limitation of this study was that the sample used in this study is the same sample as the one used to define the subtypes (Lust et al., 2022). This may limit the generalizability of the results findings. Replication of the study in a different sample is recommended. A second limitation was that the viewpoint of the children themselves was not taken into account. However, according to a meta-analysis, children tend to report more problems than parents and teachers (Huang, 2017). This may imply that the current data may even underestimate the amount of actual problems experienced by the children themselves. Therefore, in future studies we recommend to include the child perspective as well. A last limitation of this study was the lack of a control group of TD children. Data of the children were compared to reference norms (Achenbach and Rescorla, 2001). Where possible, the results from recent population-based studies were added (Van der Ende et al., 2016) to compare our findings with more recent data about mental health problems. Moreover, according to a recent review, the worldwide prevalence of mental disorders in children did not change in the past 30 years in different cultures (Polanczyk et al., 2015). However, the inclusion of an age and sex matched control group should be considered in future studies.

## 5 Conclusion

DCD is not an isolated condition, as two third of the children with DCD in our study showed symptoms of behavioral and emotional problems. Notably, behavioral and emotional problems occurred more frequently in subtypes with generalized motor problems. If children have below average performance on all subtests of a motor test and on visual perception of the VMI, in addition to borderline performance IQ, clinicians need to be extra aware of possible behavioral and emotional problems. Furthermore, the occurrence of behavioral and emotional problems is context-specific, with attention problems reported most frequently by parents, and internalizing problems most frequently by teachers. The results of our study stress the need to assess possible behavioral and emotional problems in DCD in order to guide clinicians to tailor their interventions to the needs of a child.

## Data Availability

The datasets presented in this article are not readily available to protect the confidentiality of participants. Requests to access the datasets should be directed to jessica.lust@ru.nl.
