# Supplementary material for: Developmental coordination disorder subtypes also vary in the pattern of behavioral and emotional problems
Source: Front Psychol. 2024 Nov 28;15:1418295. doi: 10.3389/fpsyg.2024.1418295 (PMC11638586; doi:10.3389/fpsyg.2024.1418295)
Supplement: Supplementary file 1 [file Table_1.docx]

Appendix A

**I**

*Comparison of the expected and the observed representation of children in CBCL category (subclinical/clinical) for each DCD subtype (Lust et al., 2022).*

|  | Subtype 1 (*n*=23)  Less Severe generalized  motor problems | | | Subtype 2 (*n*=19)  Gross motor problems | | | Subtype 3 (*n*=16)  Severe generalized  motor problems | | | Subtype 4 (*n*=14)  Fine motor problems | | |
| --- | --- | --- | --- | --- | --- | --- | --- | --- | --- | --- | --- | --- |
|  | % (sub) clinical observed | *Χ^2^*  df=1 | *p* | % (sub) clinical observed | *Χ^2^*  df=1 | *p* | % (sub) clinical observed | *Χ^2^*  df=1 | *p* | % (sub) clinical observed | *Χ^2^*  df=1 | *p* |
| CBCL % (sub)clinical expected^a^ | 7% |  |  | 7% |  |  | 7% |  |  | 7% |  |  |
| Internalizing problems | 43.5%  *n*=10 | 47.013 | <.001^*^ | 15.8%  *n*=3 | 2.255 | .133 | 50%  *n*=8 | 45.444 | <.001^*^ | 28.6%  *n*=4 | 10.007 | .002^*^ |
| Externalizing problems | 13%  *n*=3 | 1.290 | .256 | 15.8%  *n*=3 | 2.255 | .133 | 25%  *n*=4 | 7.963 | .005 | 28.6%  *n*=4 | 10.007 | .002^*^ |
| Somatic complaints | 8.7%  *n*=2 | .102 | .75 | 10.5%  *n*=2 | .363 | .547 | 12.5%  *n*=2 | .743 | .389 | 14.3%  *n*=2 | 1.142 | .285 |
| Social problems | 38.1%  *n*=8 | 31.191 | <.001^*^ | 11.8%  *n*=2^b^ | .593 | .441 | 50%  *n*=7^c^ | 39.763 | <.001^*^ | 30.8%  *n*=4^d^ | 11.282 | <.001^*^ |
| Thought problems | 19%  *n*=4 | 4.682 | .03 | 11.8%  *n*=2^b^ | .593 | .441 | 21.4%  *n*=3^c^ | 4.477 | .034 | 23.1%  *n*=3 | 5.161 | .023 |
| Attention problems | 47.8%  *n*=11 | 58.887 | <.001^*^ | 42.1%  *n*=8 | 35.968 | <.001^*^ | 56.3%  *n*=9 | 59.614 | <.001^*^ | 35.7%  *n*=5 | **Table** 17.731 | <.001^*^ |

^*^Significant p ≤.002 (Bonferoni correction); ^a^Expected according to norms CBCL; ^b^total *n*=17; ^c^total *n*= 14; ^d^total *n*= 13

**Table II**

*Comparison of the expected and the observed representation of children in TRF category (subclinical/clinical) for each DCD subtype.*

|  | Subtype 1 (*n*=23)  Less Severe generalized  motor problems | | | Subtype 2 (*n*=18)  Gross motor problems | | | Subtype 3 (*n*=16)  Severe generalized  motor problems | | | Subtype 4 (*n*=14)  Fine motor problems | | |
| --- | --- | --- | --- | --- | --- | --- | --- | --- | --- | --- | --- | --- |
|  | % (sub) clinical observed | *Χ^2^*  *df*=1 | *p* | % (sub) clinical observed | *Χ^2^*  *df*=1 | *p* | % (sub) clinical observed | *Χ^2^*  *df*=1 | *p* | % (sub) clinical observed | *Χ^2^*  *df*=1 | *p* |
| TRF % (sub)clinical expected^a^ | 7% |  |  | 7% |  |  | 7% |  |  | 7% |  |  |
| Internalizing problems | 30.4%  *n*=7 | 19.403 | <.001^*^ | 33.3%  *n*=6 | 19.174 | <.001^*^ | 37.5%  *n*=6 | 22.863 | <.001^*^ | 28.6%  *n*=4 | 10.007 | .002^*^ |
| Externalizing problems | 17.4%  *n*=4 | 3.815 | .051 | 5.6%  *n*=1 | .056 | .810 | 37.5%  *n*=6 | 22.863 | <.001* | 42.9%  *n*=6 | 27.650 | <.001^*^ |
| Somatic complaints | 4.3%  *n*=1 | .249 | .618 | 5.6%  *n*=1 | .058 | .810 | 12.5%  *n*=2 | .743 | .389 | 0%  *n*=0 | - | - |
| Social problems | 30%  *n*=6^b^ | 16.252 | <.001^*^ | 13.3%  *n*=2^d^ | .924 | .336 | 53.8%  *n*=7^f^ | 43.824 | <.001^*^ | 38.5%  *n*=5^f^ | 19.766 | <.001^*^ |
| Thought problems | 28.6%  *n*=6^c^ | 15.011 | <.001^*^ | 12.5% *n*=2^e^ | .743 | .389 | 14.3%  *n*=2^g^ | 1.142 | .285 | 7.7%  *n*=1^f^ | .010 | .922 |
| Attention problems | 13%  *n*=3 | 1.290 | .256 | 0%  *n*=0 | - | - | 31.3%  *n*=5 | 14.453 | <.001^*^ | 21.4%  *n*=3 | 4.477 | .034 |

^*^Significant *p* ≤.002 (Bonferoni correction); ^a^Expected according to norms TRF; ^b^total *n*= 20; ^c^total *n*= 21; ^d^total *n*= 15; ^e^total *n*= 16; ^f^total *n*= 13; ^e^total *n*= 14
